# The perioperative microbiome of patients undergoing rectal cancer surgery: A pilot study

**DOI:** 10.1111/codi.70397

**Published:** 2026-02-09

**Authors:** Kiedo Wienholts, Claire P. M. van Helsdingen, Henry M. Wood, Kevin Talboom, Johannes H. W. de Wilt, Daniel Bottomley, Caroline Young, Philip Quirke, Joep P. M. Derikx, Pieter J. Tanis, Roel Hompes

**Affiliations:** ^1^ Department of Surgery Amsterdam UMC Location University of Amsterdam Amsterdam The Netherlands; ^2^ Cancer Center Amsterdam, Treatment and Quality of Life Amsterdam The Netherlands; ^3^ Cancer Center Amsterdam, Imaging and Biomarkers Amsterdam The Netherlands; ^4^ Department of Paediatric Surgery, Emma Children's Hospital Amsterdam UMC Location University of Amsterdam Amsterdam The Netherlands; ^5^ Tytgat Institute for Liver and Intestinal Research Amsterdam UMC, Location University of Amsterdam Amsterdam The Netherlands; ^6^ Amsterdam Gastroenterology Endocrinology Metabolism Amsterdam The Netherlands; ^7^ Amsterdam Reproduction and Development Amsterdam The Netherlands; ^8^ Division of Pathology and Data Analytics, Leeds Institute of Medical Research at St James's University of Leeds Leeds UK; ^9^ NIHR Leeds Biomedical Research Centre Leeds UK; ^10^ Department of Surgery, Radboud University Medical Centre Radboud Institute for Health Sciences Nijmegen The Netherlands; ^11^ Department of Surgical Oncology and Gastrointestinal Surgery Erasmus MC Rotterdam The Netherlands

**Keywords:** microbiome, perioperative changes, postoperative complications, rectal cancer, surgery

## Abstract

**Aim:**

The gut microbiome plays a crucial role in health and disease, and its involvement in postoperative complications like anastomotic leakage (AL) is of growing interest. Despite substantial preclinical evidence linking microbiome alterations to surgical outcomes, human studies are scarce, particularly those exploring the perioperative dynamics of the gut microbiome beyond a single time point. This descriptive, hypothesis‐generating pilot study aims to elucidate the perioperative changes in the faecal microbiome of patients undergoing rectal cancer surgery.

**Method:**

Seventeen patients from Amsterdam University Medical Centers participated in the IMARI‐study and the IntAct‐trial between April 2020 and April 2022. All patients in these studies underwent rectal resection for malignancy with a primary anastomosis, with or without a diverting ileostomy. Samples collected included preoperative stool, intraoperative anastomotic colonic tissue and swab and postoperative stool. Bacterial DNA was extracted and analysed using 16S rRNA gene sequencing.

**Results:**

An increase in *Enterococcus* and *Streptococcus* was observed postoperatively compared to preoperative and intraoperative samples. Postoperative samples showed a significant decrease in alpha diversity compared to preoperative and intraoperative samples. Beta diversity analysis revealed distinct clustering of postoperative stool and ileostomy samples. Preoperative oral antibiotics significantly altered the intraoperative microbiome composition and reduced postoperative alpha diversity.

**Conclusion:**

This pilot study reveals significant perioperative shifts in the gut microbiome of rectal cancer patients. These findings underscore the importance of considering microbiome dynamics perioperatively when designing and interpreting studies that correlate the microbiome with clinical outcomes. However, the conclusions should be viewed as preliminary and require confirmation in larger studies, including causal relation, to postoperative outcomes.

**What does this paper add to the literature?**

This pilot study characterizes perioperative microbiome dynamics in rectal cancer surgery using preoperative, intraoperative and postoperative samples, addressing limited human data. It demonstrates postoperative blooms of *Enterococcus*/*Streptococcus*, reduced alpha diversity, antibiotic‐associated shifts and distinct profiles for ileostomy effluent versus stool, highlighting methodological confounders that future outcome‐focused studies should control for.

## INTRODUCTION

The gut microbiome has become an intriguing and rapidly expanding field of research [1]. Several hypotheses have been formulated regarding the role of the gut microbiome in relation to different disease entities. In particular, the role as a potential driver for anastomotic leakage (AL) has sparked interest, as AL remains a devastating complication following colorectal cancer surgery, with an incidence reaching up to 20% in rectal cancer surgery [2]. Several studies have linked the intestinal microbiome to AL, and the first study exploring this was performed in 1955 [3, 4, 5]. Subsequent research pushed the microbial aspect of AL to the background. However, the arrival and wide availability of new techniques, such as 16S rRNA analysis, has reactivated research interest in exploring the role of the microbiome in AL.

The gut microbiota plays an important role in metabolic regulation, immune modulation and protection against pathogens [6]. Preclinical studies have demonstrated how intestinal bacteria dynamically respond to host environment alterations. Factors such as tissue injury, ischaemia, surgical interventions, dietary modifications and perioperative antibiotic regimens have been shown to profoundly shift microbiome composition in experimental rat models [7]. Importantly, distinct microbiome profiles have been identified in both human and murine models, correlating with favourable and unfavourable healing outcomes of anastomoses [7, 8]. Yet, most of these microbiome studies have been conducted in animals and the extent to which these findings translate to the human microbiome remains largely unexplored.

Some human‐based studies have demonstrated the efficacy of preoperative intestinal decontamination using oral and intravenous antibiotics in reducing the incidence of surgical complications, including infections or AL and analysed the microbiome of such patients [9, 10]. However, most of these studies only analysed the microbiome at a single timepoint—before, during, or after surgery—and could not capture the dynamics of the microbiota surrounding surgery, influenced by various perioperative factors. Therefore, the primary objective of this exploratory pilot study is to elucidate the dynamics of the preoperative, intraoperative and postoperative human faecal microbiome in a cohort of patients undergoing rectal cancer surgery. The present study was designed as a descriptive, hypothesis‐generating pilot and the sampling window was enabled by co‐enrolment in two ongoing studies: the IMARI‐study and IntAct‐trial. The aim here was not to link microbiome features to clinical outcomes, but to document perioperative patterns that can inform the design of larger, outcome‐linked studies.

## METHOD

### Population

The samples used in this study were all obtained from patients (*n* = 17) who underwent surgery at the Amsterdam University Medical Centers (AUMC) and were co‐enrolled in both the IMARI‐study (a multicentre, prospective, clinical effectiveness cohort study) and the IntAct‐trial (a prospective, unblinded, parallel‐group, multicentre, European, randomized controlled trial) between April 2020 and April 2022 [11, 12]. The IMARI‐study is designed to evaluate the efficacy of a multi‐interventional program in reducing AL and improving the management of AL. This trial has been open for inclusion since January 2020 and the primary endpoint of this study is the one‐year anastomotic integrity rate, with a microbiome sub‐study as part of this trial. The IntAct trial compares restorative rectal cancer surgery with intra‐operative fluorescence angiography (IFA) against standard care (surgery without IFA). The primary outcome is the difference in clinical AL rate at 90 days post‐surgery between the two groups and a microbiome sub‐study is part of this trial. All patients in these studies underwent rectal resection for malignancy with a primary anastomosis, with or without a diverting ileostomy. Standard ERAS elements (single preoperative IV antibiotic prophylaxis, early mobilization and early oral intake) were applied according to the local ERAS protocol of the AUMC. No standardized prehabilitation program was in place during the study period. Patients routinely received a single pre‐operative parenteral dose of 2000 mg cefazoline and 500 mg metronidazole, and oral mechanical bowel preparation with polyethylene glycol (Moviprep®) was administered pre‐operatively. Additionally, three patients received preoperative oral antibiotics following the local protocol at one of the AUMC locations. Specifically, they received 10 mL of SDD solution three times daily for 3 days prior to surgery. Each 10‐mL dose contained 100 mg of colistin, 80 mg of tobramycin and 2000000 IU of nystatin. Transanal Total Mesorectal Excision with a laparoscopic abdominal approach and end‐to‐end anastomosis was performed in all patients.

### Sample collection

For patients who gave additional consent for storage of samples in the IMARI biobank, the following samples were collected and analysed in this study [12]: (1) stool sample pre‐operatively before the start of antibiotics or mechanical bowel preparation; (2) anastomotic doughnut (colonic side) during surgery (this is the ring of colon that is cut by a circular stapler during the construction of the anastomosis); (3) rectal swab from the anastomotic site during surgery; (4) stool sample on postoperative day 4. To minimize post‐sampling microbial changes, all samples were stored at –80°C without buffer in a sterile Eppendorf tube as soon as possible after a maximum of 24 h in a refrigerator. Bacterial DNA was isolated from all samples and was subsequently analysed using sequencing of the amplified 16S rRNA genes.

### DNA extraction and 16S rRNA sequencing

DNA was extracted using the QIAamp DNA mini kit. 20 ng of DNA per sample was prepared for V4 16S rRNA sequencing using protocols from the Earth Microbiome Project [13]. Samples were pooled and sequenced alongside samples from other projects on an Illumina NextSeq 2500 2 × 150 bp run.

### Bioinformatic analysis

Sequence reads were trimmed of adapters using Cutadapt [14] before being imported into the QIIME2 framework [15] for further processing. Reads were filtered, pairs merged, and assigned to sequence variants (ASVs) using DADA2 [16]. Alpha (within sample) diversity was measured using the Shannon index [17]. Beta (between sample) diversity was measured using Bray‐Curtis distances [18]. Associations between beta diversity and sample metadata were calculated using Adonis PERMANOVA analysis [19], with *p*‐values calculated from 1000 iterations. Taxa were assigned using the q2‐feature classifier BLAST plugin, aligning to the SILVA database [20, 21, 22]. Where appropriate, groups were compared using the Mann–Whitney test [23]. Statistical analyses were performed by Henry M. Wood, who had the appropriate statistical expertise.

### Ethical approval

This study has been approved by the Medical Ethical Committee and Biobank committee of the Amsterdam UMC, location AMC. The protocol is registered with the Dutch Central Committee on Research Involving Human Subjects (NL67600.018.18) and has been submitted to the www.onderzoekmetmensen.nl/en database (NL‐OMON26456 & NL‐OMON55903). Written informed consent was obtained from all participants in this study, and separate consent was obtained for storage of samples in the IMARI biobank. This study adhered to the STROBE Statement for cohort studies [24].

## RESULTS

### Study population

In this pilot study, 17 patients from the AUMC participating in both the IMARI‐study and IntAct‐trial were included. The majority (71%) of patients were male. Mean age at the time of the index surgery was 59 years (SD 12) with a mean BMI of 25 kg/m^2^ (SD 4). Baseline characteristics are displayed in Table 1. Most patients (82%) did not receive neoadjuvant (chemo)radiation and 6 patients (35%) had previously undergone endoluminal excision. Three patients (18%) received oral antibiotics preoperatively, and an ileostomy was constructed in 7 patients (41%).

**TABLE 1:** Baseline characteristics.

Variable	Total counts (%)^a^ *N* = 17
Sex, male	12 (71)
Age at time of index surgery, mean ± SD (years)	59 ± 12
BMI, mean ± SD (kg/m^2^)	25 ± 4
Tumour from anorectal junction on MRI, mean ± SD (mm)	31 ± 20
ASA classification	
ASA I	4 (24)
ASA II	11 (65)
ASA III	2 (12)
ASA IV	0
Active smoker	3 (18)
Neoadjuvant therapy	
None	14 (82)
Short‐course radiotherapy (5 × 5 Gy) only	1 (6)
Chemoradiotherapy	2 (12)
Previous endoluminal excision	6 (35)
Preoperative oral antibiotics	3 (18)
Construction of anastomosis	
Double purse‐string, single‐stapled	15 (88)
Double stapled	0
Hand‐sewn	2 (12)
Double loop ileostomy during surgery	7 (41)

Abbreviations: ASA, American Society of Anaesthesiologists; BMI, body mass index; SD, standard deviation.

^a^ Variables are *n* (%) unless otherwise indicated.

The microbial composition was successfully determined in 13 out of 14 preoperative faeces samples, 12 out of 16 intraoperative swab samples, 17 out of 17 intraoperative doughnut samples and 11 out of 13 postoperative faeces samples. In some samples, the microbial composition could not be reliably determined due to insufficient amounts of bacterial DNA. Additionally, faecal samples were not collected from three patients pre‐operatively due to late inclusion, from four patients postoperatively due to early discharge from the hospital, and collection of one intra‐operative swab was omitted.

### Bacterial composition perioperatively

**FIGURE 1:**
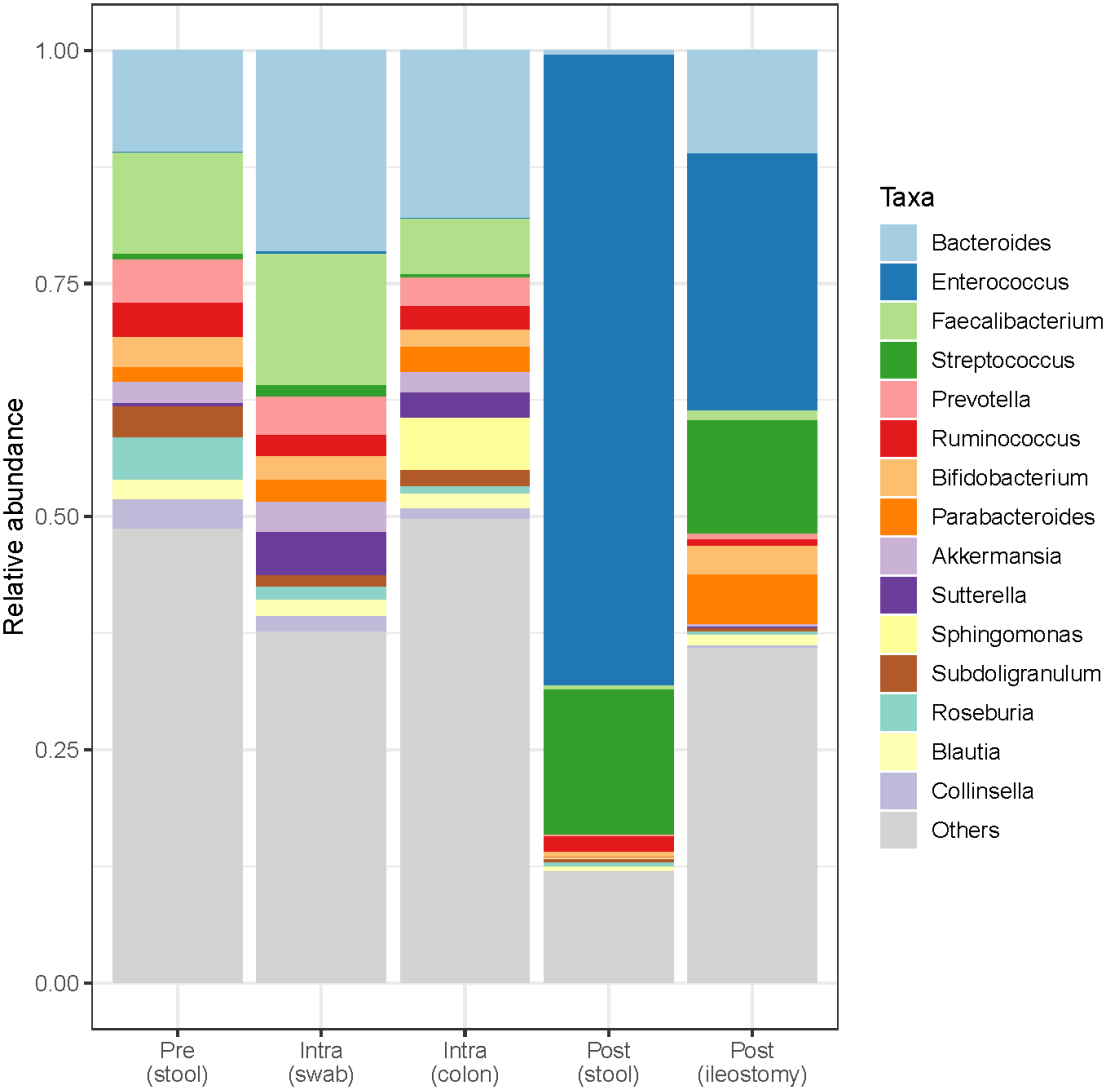
Mean relative abundance preoperatively, intraoperatively and postoperatively. Pre (stool) = preoperative faeces from stool; Intra (swab) = intraoperative swab anastomotic site; Intra (colon) = intraoperative doughnut colon; Post (stool) = postoperative faeces from stool; Post (ileostomy) = postoperative faeces from ileostomy.

Relative abundances of the 15 most abundant genera showed great similarity between preoperative and intraoperative samples (Figure 1). *Bacteroides* was the most abundant genus in the preoperative faeces, intraoperative swabs and intraoperative doughnuts (mean relative abundance of 11% vs. 22% vs. 18%, respectively), and *Faecalibacterium* was the second most abundant genus for these timepoints (mean relative abundance = 11% vs. 14% vs. 6%, respectively). The genus *Sphingomonas* was only present in the intraoperative doughnut samples (6%) and absent in the other samples. The most significant change in bacterial composition was seen postoperatively with a shift to *Enterococcus* and *Streptococcus* as the most abundant genera. Mean relative abundances of *Enterococcus* and *Streptococcus* were higher in postoperative stool samples (68% and 16%, respectively) compared to postoperative ileostomy samples (28% and 12% respectively).

### Alpha and beta diversity perioperatively

**FIGURE 2:**
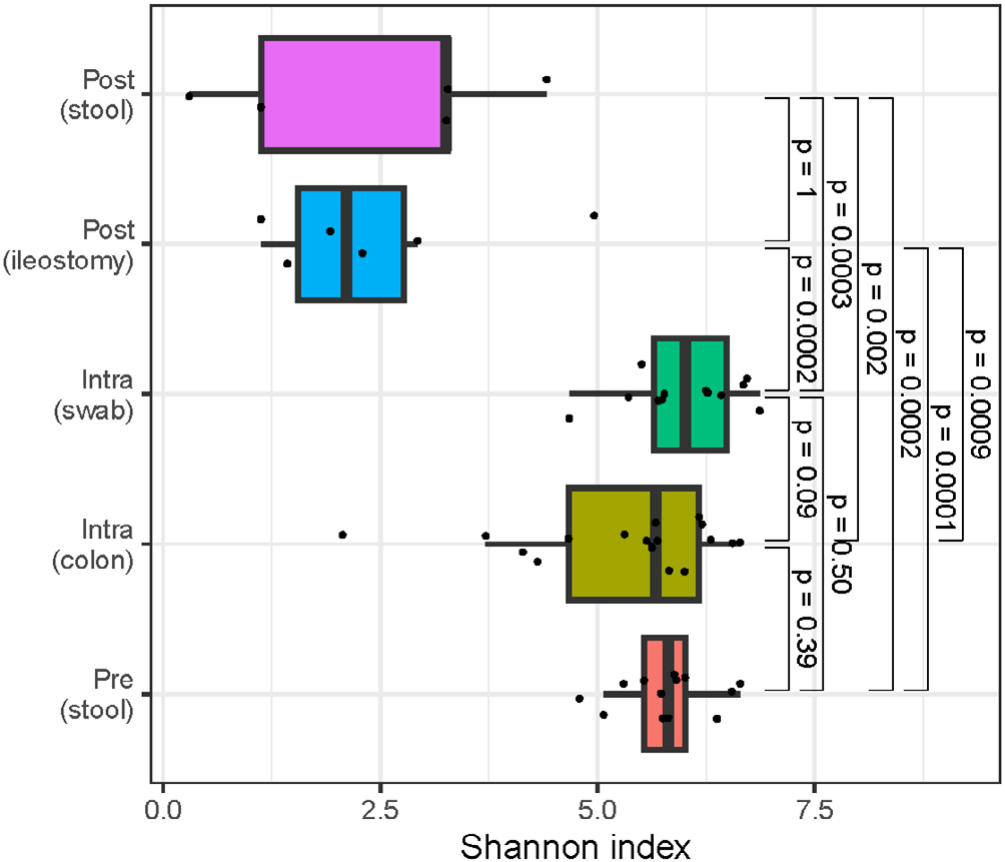
Shannon index preoperatively, intraoperatively and postoperatively. Pre (stool) = preoperative faeces from stool; Intra (colon) = intraoperative doughnut colon; Intra (swab) = intraoperative swab anastomotic site; Post (ileostomy) = postoperative faeces from ileostomy; Post (stool) = postoperative faeces from stool.

**FIGURE 3:**
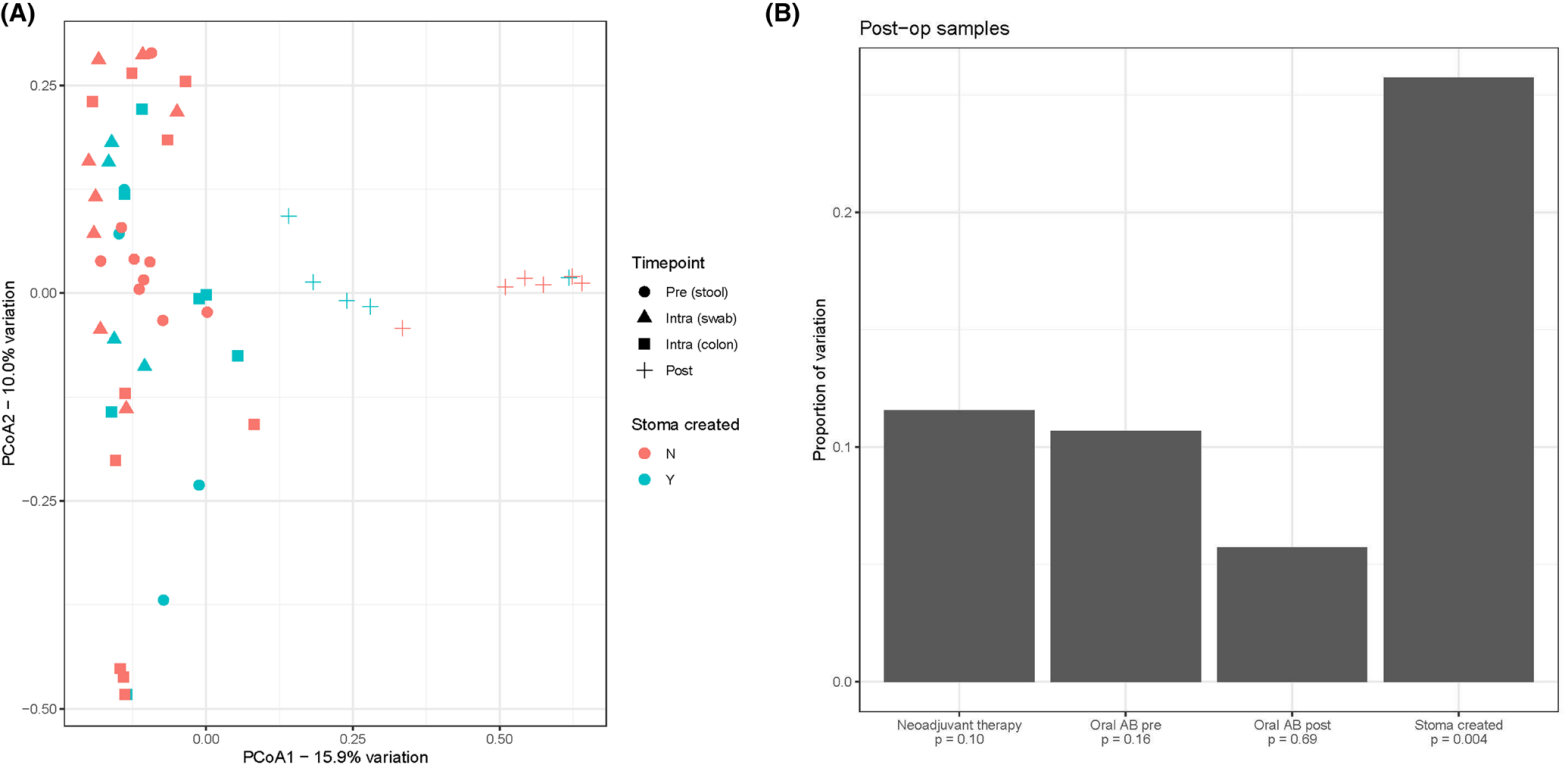
(A) PCoA of all timepoints and stoma creation. Pre (stool) = preoperative faeces from stool; Intra (swab) = intraoperative swab anastomotic site; Intra (colon) = intraoperative doughnut colon; Post = postoperative faeces. (B) Adonis analysis of postoperative samples. Post‐op = postoperative; Oral AB post = postoperative oral antibiotics; Oral AB pre = preoperative oral antibiotics. N, no; PCoA, principal coordinate analysis; Y, yes.

Alpha diversity was significantly decreased in postoperative samples, compared to preoperative and intraoperative samples (Figures 2 and S1A,B). A clear distinction of alpha diversity between postoperative stool and ileostomy samples could not be observed. Principal coordinates analyses (PCoA) were used to assess beta diversity and revealed a clear clustering of postoperative samples compared to the other samples (Figure 3A). In Figure 3B, we specifically highlight that postoperative ileostomy samples had significant differences in beta diversity (*p* = 0.004) compared to postoperative faeces samples.

To ensure that sample type did not confound the analyses, we investigated the proportion of beta‐diversity attributable to patient, timepoint, and sample type (Figure S2). Although sample type was significant, it was associated with considerably less than 5% of the variation, considerably less than timepoint or patient.

### Impact of preoperative oral antibiotics and neoadjuvant therapy on intraoperative microbiome

**FIGURE 4:**
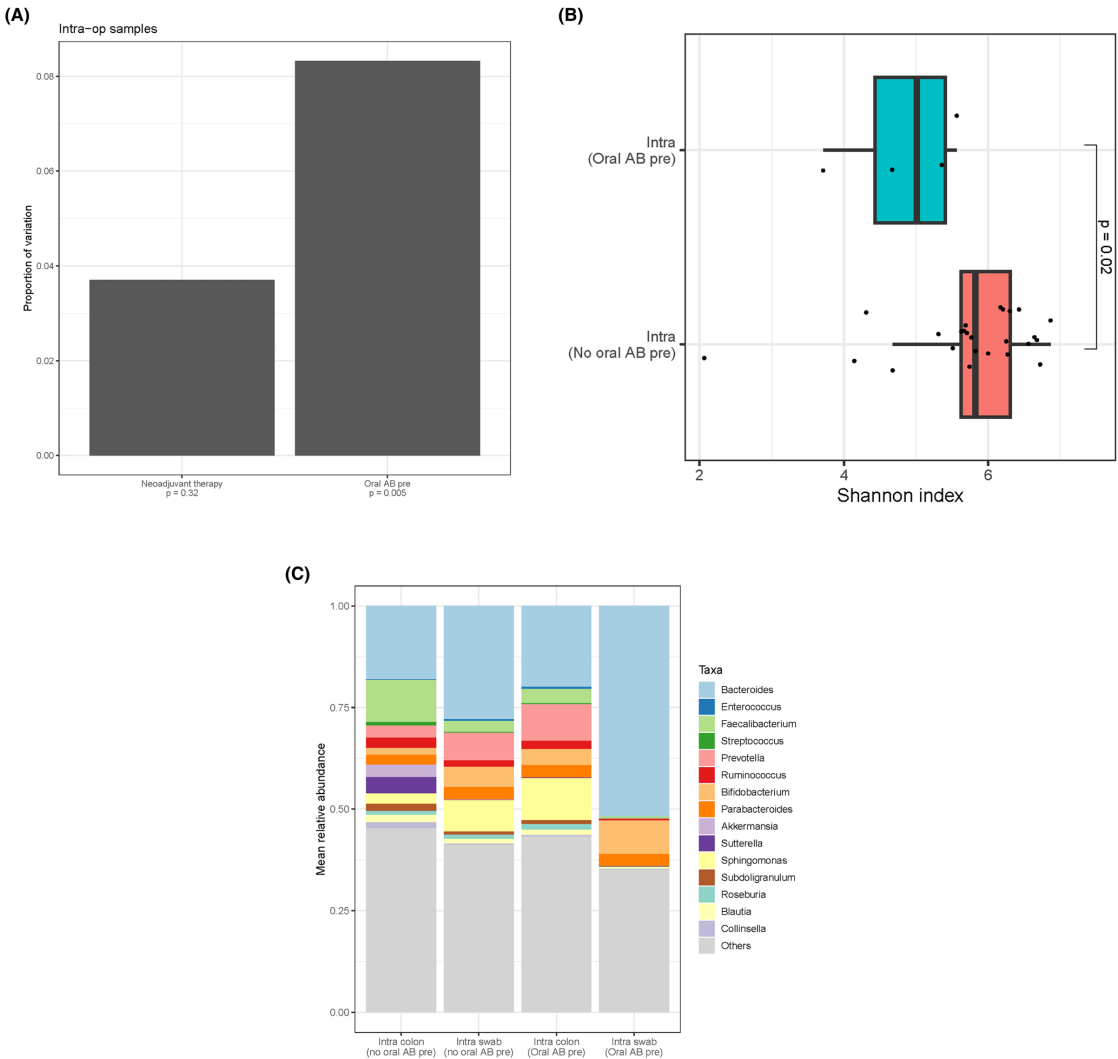
(A) Adonis analysis of intraoperative samples. Intra‐op = intraoperative; Oral AB pre = preoperative oral antibiotics; (B) Shannon index postoperatively. Intra = intraoperative samples (doughnut colon + swab anastomotic site); Oral AB pre = preoperative oral antibiotics; (C) Comparison of mean relative abundances intraoperatively and preoperative oral antibiotics. Intra colon = intraoperative doughnut colon; Intra swab = intraoperative swab anastomose site; Oral AB pre = preoperative oral antibiotics.

Preoperative oral antibiotic administration significantly altered the beta diversity of intraoperative samples (*p* = 0.005), while neoadjuvant therapy did not (*p* = 0.32; Figure 4A). Alpha diversity was significantly decreased in patients who received oral antibiotics compared to patients who did not receive oral antibiotics (*p* = 0.02; Figures 4B and S1C,D). Relative abundances of intraoperative samples were compared between patients that received oral antibiotics preoperatively and those who did not in Figure 4C. Other variables such as neo‐adjuvant therapy, AL and postoperative oral antibiotics administration did not significantly alter the beta diversity of the corresponding samples.

## DISCUSSION AND CONCLUSIONS

This is the first study to describe the dynamics of the perioperative human faecal microbiome in patients undergoing rectal cancer surgery. A remarkable bloom in *Enterococcus* and *Streptococcus* was observed postoperatively, together with a significant decrease in alpha diversity. The microbial composition of postoperative ileostomy samples differed significantly from that of postoperative stool samples. Furthermore, preoperative oral antibiotics had a substantial impact on the intraoperative microbiome composition and postoperative alpha diversity, although only a small number of patients received them, limiting broad conclusions. These signals should be interpreted as hypothesis‐generating rather than generalisable effects, consistent with prior perioperative reports but requiring testing in larger, outcome‐linked cohorts.

The postoperative bloom in *Enterococcus* and *Streptococcu*s observed in this study has been reported in previous research, but in different patient cohorts [8, 25, 26, 27]. For instance, one study analysed faecal samples at various time points perioperatively in patients undergoing both colon and rectal surgery and found significant alterations in microbial composition up to ten days postoperatively, including a bloom in *Enterococcus*, *Lactobacillus* and *Streptococcus*, with a return towards baseline by 30–180 days [25]. Another study in patients undergoing colonic surgery identified *Enterococcus* as dominant on the first postoperative day [8], and increases by day seven have been described after surgery in cohorts including both colon and rectal surgery [26, 27]. In our study, which focused solely on rectal cancer patients undergoing rectal resection, we observed a similar increase in both *Enterococcus* and *Streptococcus* on postoperative day four. It is conceivable that these postoperative microbial changes begin on the first postoperative day and persist until around 10 days postoperatively before gradually returning to baseline levels, as described by Nalluri‐Butz et al. [25]. These findings are substantial because such microbial alterations may be associated with adverse postoperative outcomes. *Enterococcus* and *Streptococcus* are known pathobionts linked to AL in animal studies, where a 500‐fold increase in Enterococcus was observed in postoperative colon tissue of rats that developed AL [28, 29, 30, 31]. Mechanistically, *Enterococcus* has been implicated in the development of AL through the production of collagenases, leading to tissue degradation [32, 33]. Given that AL mostly occurs in the early postoperative period, the early postoperative increase in *Enterococcus* and *Streptococcus* calls for further investigation in larger human studies to correlate these microbial changes with clinical outcomes, as current microbiome studies lack the power to adequately address this issue.

In general, disturbances in microbiome composition can trigger dysbiosis and favour the proliferation of typically low‐abundant pathobionts, thereby promoting the development of surgical complications. *Enterococcus* and *Streptococcus* are facultative anaerobes and typically low‐abundant pathobionts, while *Bacteroides* and *Faecalibacterium* are obligate anaerobes and crucial for maintaining the environmental stability of the intestinal tract [34, 35]. An increase in oxygen levels in the colorectal lumen during surgery, along with perioperative antibiotics regimens and tissue injury, may explain the shift from *Bacteroides* and *Faecalibacterium* as dominant strains pre‐operatively to *Enterococcus* and *Streptococcus* postoperatively. This major compositional shift from preoperative to postoperative microbiome, acting as a trigger for dysbiosis, presents an intriguing research area with an urgent need for larger human studies. We highlight that this compositional shift should be interpreted as a perioperative signature consistent with dysbiosis; whether it is beneficial or harmful for healing cannot be determined from this pilot and requires outcome‐linked studies. Preclinical work has implicated collagenolysis and impaired wound repair in anastomotic failure [28, 29, 30, 31, 32, 33]. However, those mechanisms are strain‐ and function‐specific and cannot be tested with the present 16S dataset; therefore we deliberately avoid functional inference.

Alpha diversity was significantly decreased postoperatively compared to preoperatively, consistent with findings from the limited human literature [25, 27, 36]. Nalluri‐Butz et al. observed no decrease after colonoscopy with mechanical bowel preparation alone (without antibiotics), suggesting that antibiotic exposure is a principal driver [25]. Our results are compatible with a rapid, early postoperative drop in alpha diversity, likely reflecting the combined effects of prophylactic antibiotics, surgical stress and perioperative perturbations. Given the sample size and study goal, we do not attempt to subdivide individual contributors or to link alpha diversity to outcomes. Instead, we view the alpha diversity decrease as a reproducible perioperative feature that merits time‐course mapping and clinical correlation in larger cohorts.

Our results reveal a distinct separation in microbial composition between postoperative ileostomy samples and stool samples. One prior study compared microbiota in high‐ versus low‐output ileostomies after rectal cancer surgery and found differences in *Bacteroides* abundance [37]. This study also found that the phylum *Firmicutes*, which includes the genera *Enterococcus* and *Streptococcus*, was the dominant strain in both ileostomy groups, which is in line with our findings. Separation between ileostomy effluent and stool is not surprising, but it is practically important to consider its magnitude and analytical consequence in perioperative research. Combining ileostomy and stool in a single analysis may inflate heterogeneity and obscure antibiotic‐associated signals. Therefore, we recommend stratified analysis and substance‐specific sampling frames in surgical microbiome studies and trials.

Preoperative oral antibiotics significantly altered the intraoperative microbiome and were associated with a lower postoperative alpha diversity compared with patients who did not receive oral antibiotics. This is consistent with surgical cohorts receiving mechanical bowel preparation plus oral/IV antibiotics, in whom compositional shifts are observed [25]. In our study, only three patients received oral antibiotics. As such, the signal should be viewed as hypothesis‐generating.

This study has several limitations that must be acknowledged. Firstly, and most importantly, the small sample size limited the statistical power and hindered any robust correlations with clinical outcomes. This limitation also prevented matching of patients based on key variables such as age, sex, or comorbidities, which could confound the interpretation of microbiome changes. In a follow‐up study, we plan to include a significantly larger cohort of IMARI‐study patients to validate the findings of this pilot study and to investigate the relationship between dynamic microbiome changes and clinical outcomes. Secondly, the study did not account for the effect of prior endoluminal excision and dietary influences on the microbiome, which are known to significantly affect microbial composition and diversity. This could have introduced variability in the results unrelated to the surgery and perioperative factors alone. Lastly, short‐read 16S limits strain/functional resolution; however, its feasibility and cost‐efficiency make it suitable for screening and hypothesis generation. Outcome‐linked testing with absolute quantification and shotgun metagenomics is planned in future studies.

In conclusion, this study elucidates the dynamics of the human faecal microbiome—preoperative, intraoperative and postoperative—in patients undergoing rectal cancer surgery. Our findings highlight a significant postoperative bloom in *Enterococcus* and *Streptococcus*, alongside a marked decrease in alpha diversity. We also observed distinct differences in the microbial composition of postoperative ileostomy versus stool samples, which should be considered in future studies. Moreover, preoperative oral antibiotics significantly influenced the intraoperative microbiome composition and postoperative diversity. This study underscores the necessity for larger, more comprehensive microbiome studies in humans. Outcome‐linked, mechanistic and large‐scale analyses are planned within the IMARI‐study to test clinically relevant hypotheses. Until such data are available, the present findings should be interpreted as foundational and hypothesis‐generating, offering an empirical baseline for the next phase of perioperative microbiome research in rectal cancer surgery. Overall, these perioperative signals offer a practical framework for sampling and analysis and will inform prospectively powered, outcome‐linked investigations.

## AUTHOR CONTRIBUTIONS

All authors made substantial contribution to the study and the manuscript. All authors gave final approval of the version of the manuscript to be published. **Kiedo Wienholts:** Study conception design; Data acquisition; Data analysis and interpretation; Drafting the article; Critical revision for intellectual content. **Claire P.M. van Helsdingen:** Data acquisition; Drafting the article; Critical revision for intellectual content. **Henry M. Wood:** Study conception design; Data analysis and interpretation; Drafting the article; Critical revision for intellectual content. **Kevin Talboom:** Data acquisition; Drafting the article; Critical revision for intellectual content. **Johannes H.W. de Wilt:** Data analysis and interpretation; Drafting the article; Critical revision for intellectual content. **Daniel Bottomley:** Data acquisition; Drafting the article; Critical revision for intellectual content. **Caroline Young:** Data analysis and interpretation; Drafting the article; Critical revision for intellectual content. **Philip Quirke:** Data analysis and interpretation; Drafting the article; Critical revision for intellectual content. **Joep P.M. Derikx:** Study conception design; Data analysis and interpretation; Drafting the article; Critical revision for intellectual content. **Pieter J. Tanis:** Study conception design; Data analysis and interpretation; Drafting the article; Critical revision for intellectual content. **Roel Hompes:** Study conception design; Data analysis and interpretation; Drafting the article; Critical revision for intellectual content.

## FUNDING INFORMATION

The IMARI‐study is an investigator‐initiated study funded by the Dutch Cancer Society (KWF) and third‐party funding by B. Braun Surgical, S. A and Stryker European Operations B.V. with no influence on protocol writing and no access to data. Microbiome analyses at the University of Leeds were funded by CRUK Grand Challenge OPTIMISTICC (C10674/A27140). HW, DB, CY and PQ are supported in part by the National Institute for Health and Care Research (NIHR) Leeds Biomedical Research Centre (BRC) (NIHR203331). The views expressed are those of the author(s) and not necessarily those of the NHS, the NIHR or the Department of Health and Social Care.

## CONFLICT OF INTEREST STATEMENT

Roel Hompes receives grants and materials from Stryker European Operations B.V. and consultancy fees from Applied Medical. The funders had no role in the design and conduct of the study; collection, management, analysis and interpretation of the data; preparation, review, or approval of the manuscript; and decision to submit the manuscript for publication. Kiedo Wienholts, Claire P.M. van Helsdingen, Henry M. Wood, Kevin Talboom, Johannes H.W. de Wilt, Daniel Bottomley, Caroline Young, Philip Quirke, Joep P.M. Derikx and Pieter J. Tanis, have no conflicts of interest to disclose.

## ETHICS STATEMENT

This study is approved by the Medical Ethical Committee and Biobank committee of the Amsterdam UMC, location AMC.

## PATIENT CONSENT STATEMENT

Written informed consent was obtained from all participants in this study and separate consent was obtained for storage of samples in the IMARI biobank.

## CLINICAL TRIAL REGISTRATION

The protocol is registered by the Dutch Central Committee on Research Involving Human Subjects (NL67600.018.18) and is submitted to the www.onderzoekmetmensen.nl/en database (NL‐OMON26456 & NL‐OMON55903).

## Supporting information


Figure S1.

Data S1.

## Data Availability

The data that support the findings of this study are available on request from the corresponding author. The data are not publicly available due to privacy or ethical restrictions.
