# Supplementary material for: The perioperative microbiome of patients undergoing rectal cancer surgery: A pilot study
Source: Colorectal Dis. 2026 Feb 9;28(2):e70397. doi: 10.1111/codi.70397 (PMC12886600; doi:10.1111/codi.70397)
Supplement: Supplementary file 1 — Figure S1. [file CODI-28-0-s002.docx]

**Supplementary figure S1A: Simpson index preoperatively, intraoperatively, and postoperatively**

**
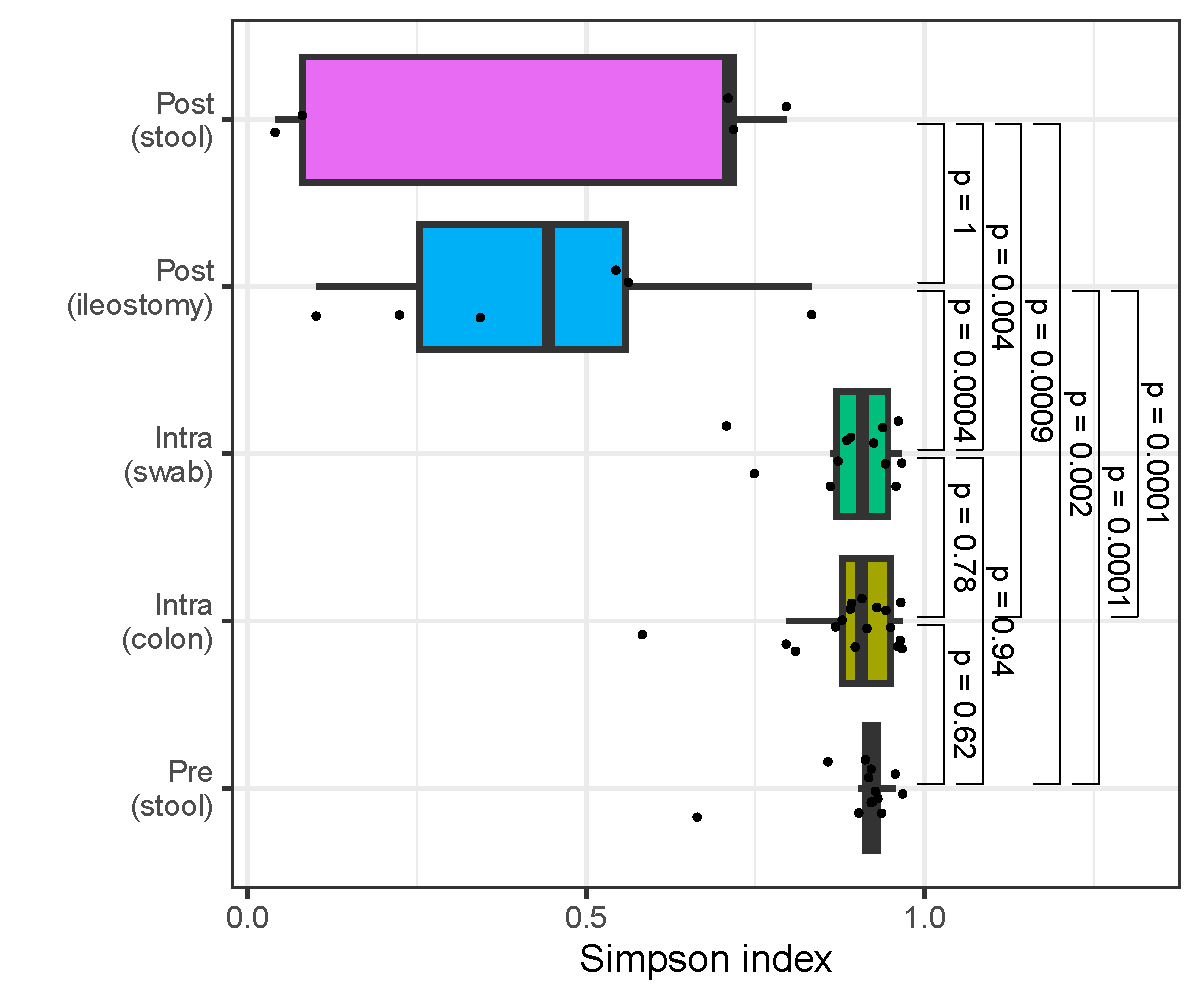
**

Post (stool) = postoperative feces from stool. Post (ileostomy) = postoperative feces from ileostomy. . Intra (swab) = intraoperative swab anastomotic site. Intra (colon) = intraoperative donut colon. Pre (stool) = preoperative feces from stool.

**Supplementary figure S1B: Chao1 index preoperatively, intraoperatively, and postoperatively**

**
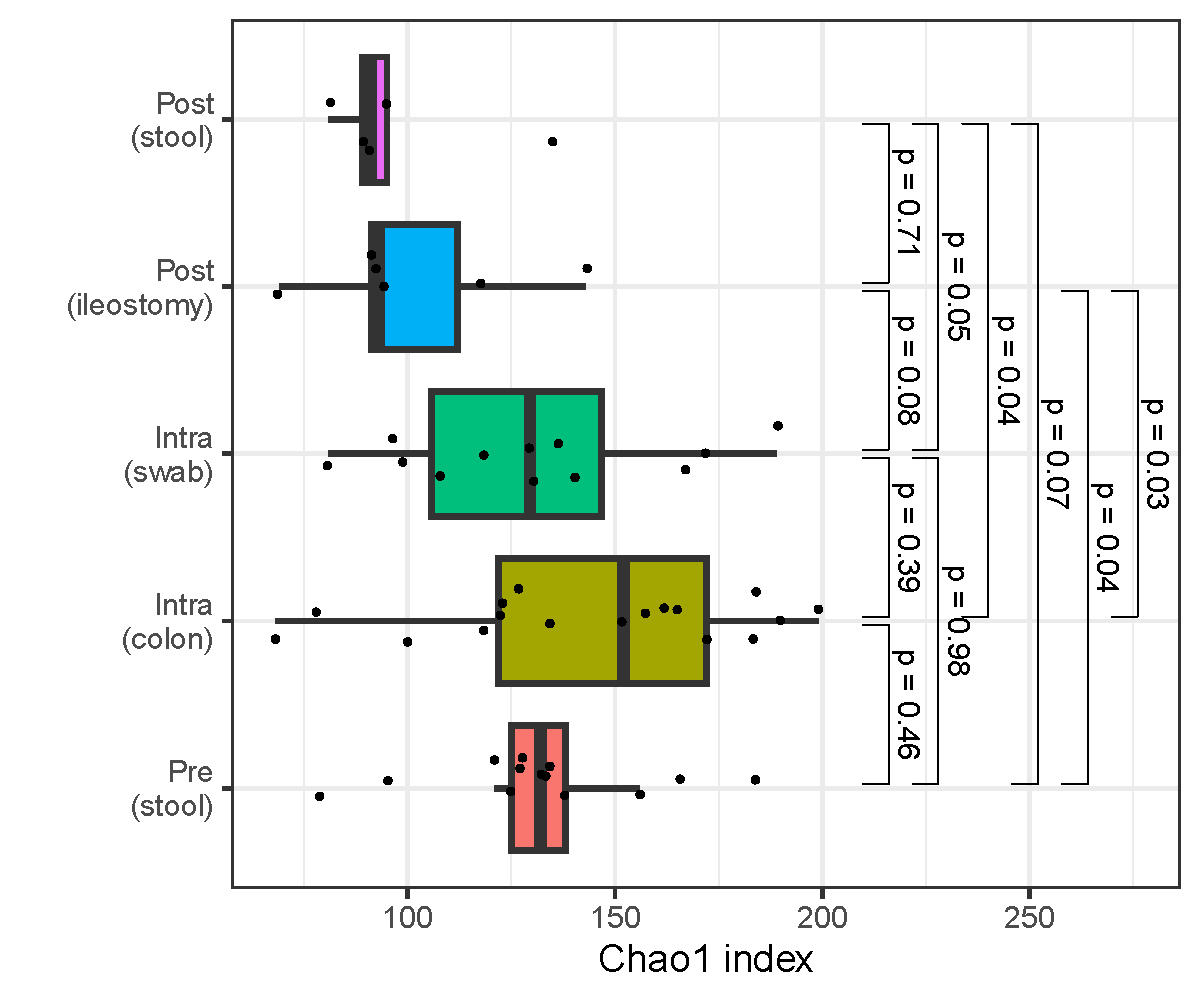
**

Post (stool) = postoperative feces from stool. Post (ileostomy) = postoperative feces from ileostomy. . Intra (swab) = intraoperative swab anastomotic site. Intra (colon) = intraoperative donut colon. Pre (stool) = preoperative feces from stool.

**Supplementary figure S1C: Simpson index postoperatively**

**
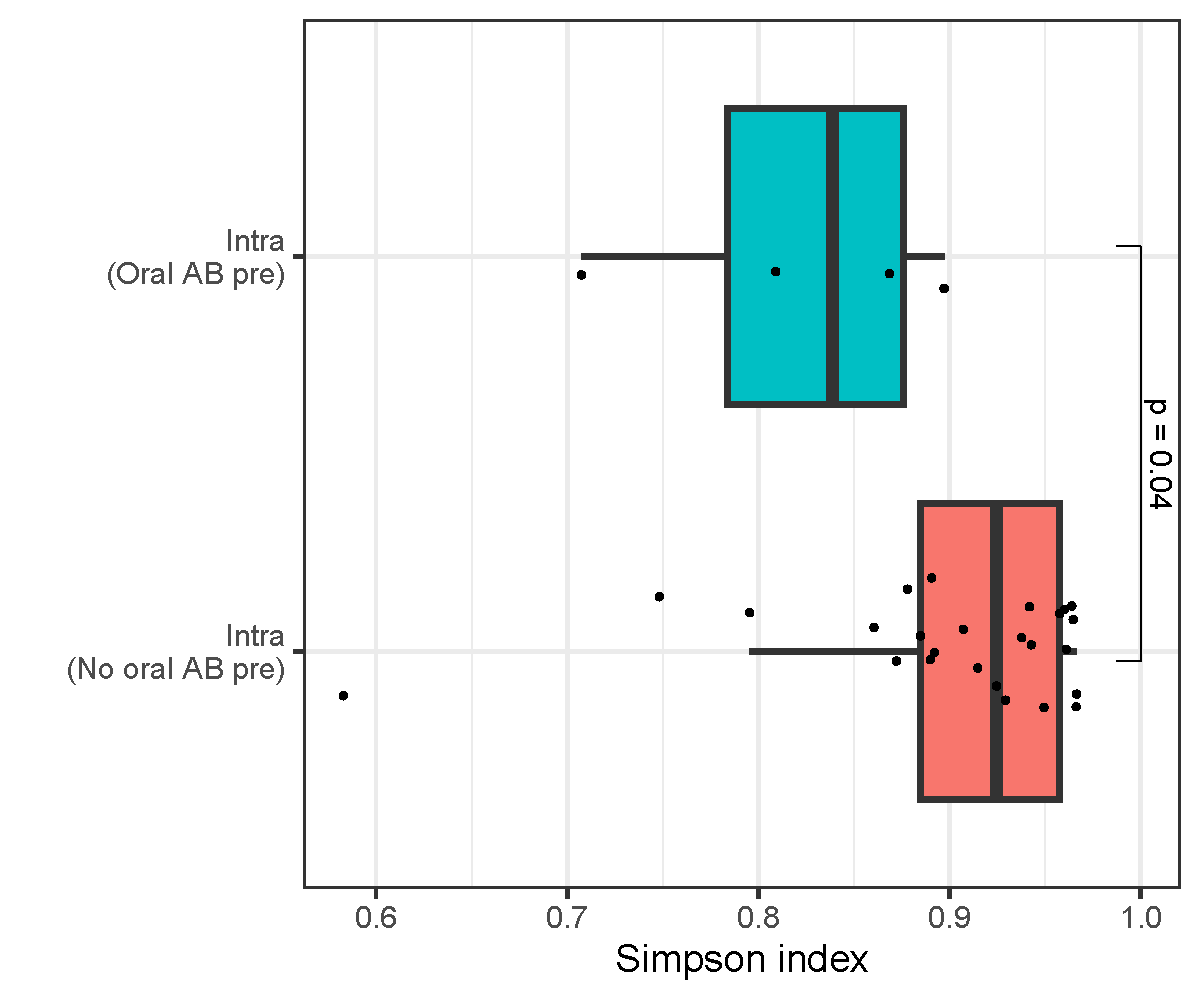
**

Intra = intraoperative samples (donut colon + swab anastomotic site). Oral AB pre = preoperative oral antibiotics.

**Supplementary figure S1D: Chao1 index postoperatively**


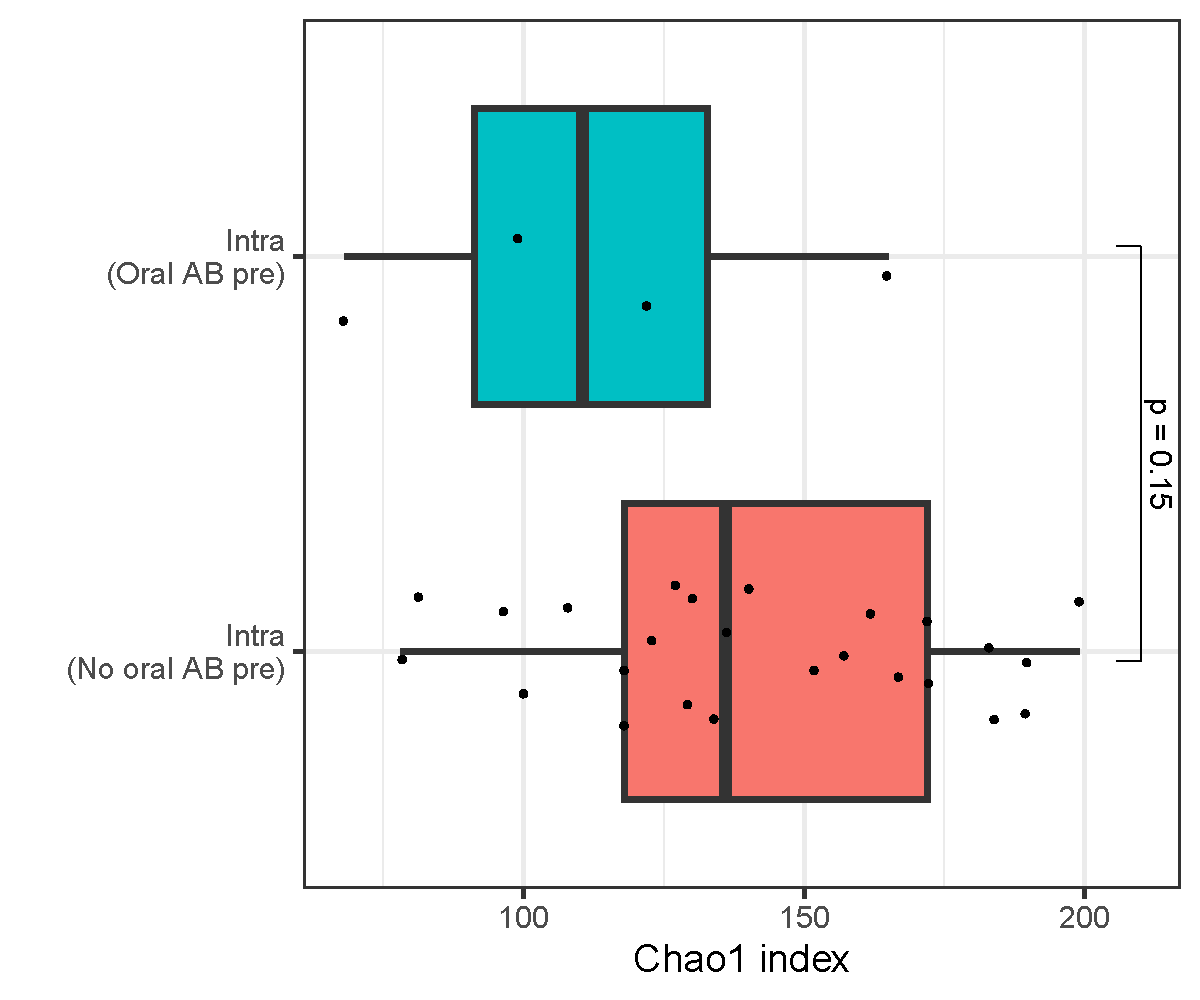


Intra = intraoperative samples (donut colon + swab anastomotic site). Oral AB pre = preoperative oral antibiotics.

**Supplementary figure S2. Adonis analysis of all samples with proportion of variance explained by different patients, sample types, and timepoints**

**
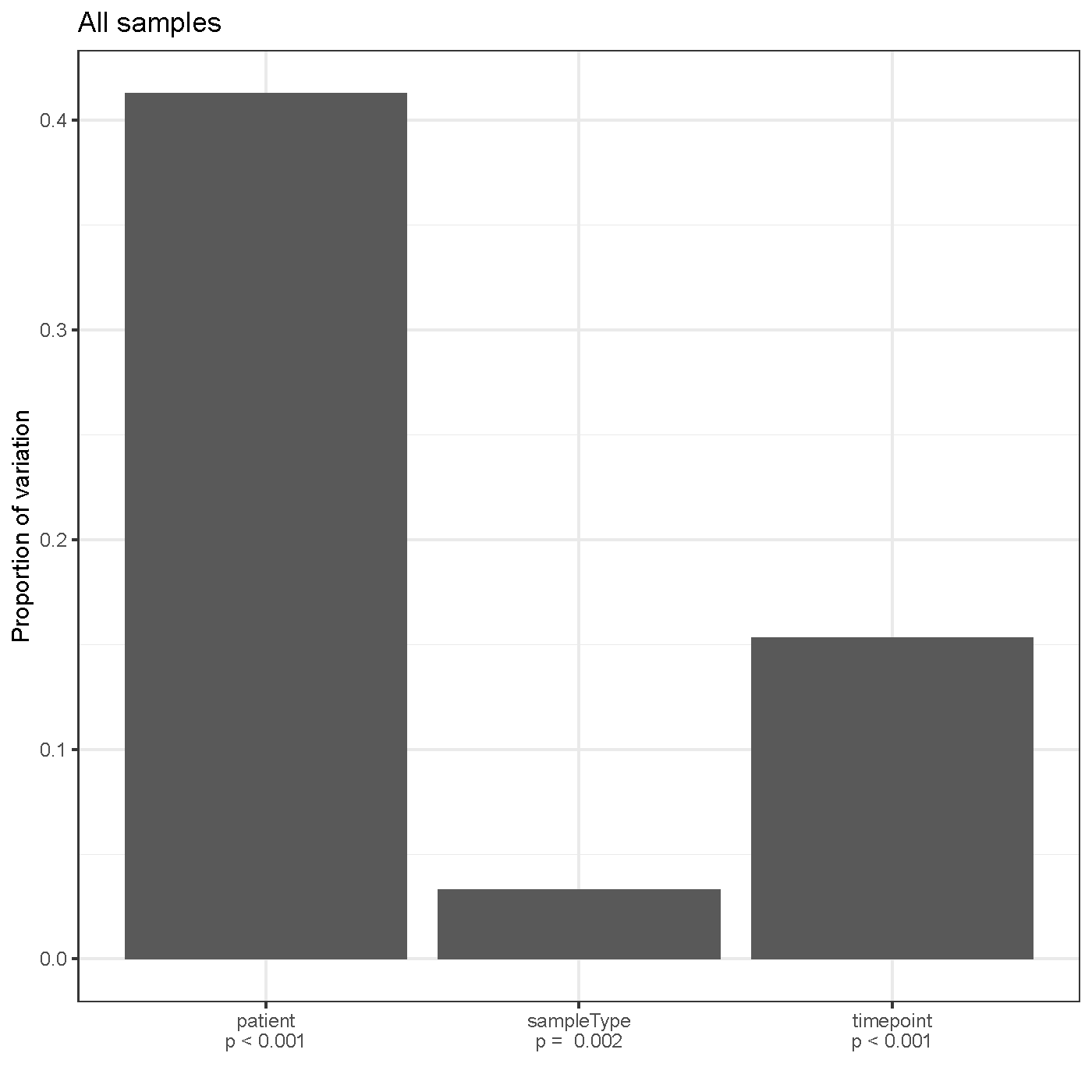
**
